# Inhibition of *Arenaviridae* nucleoprotein exonuclease by bisphosphonate

**DOI:** 10.1107/S2052252522005061

**Published:** 2022-05-28

**Authors:** Thi Hong Van Nguyen, Elsie Yekwa, Barbara Selisko, Bruno Canard, Karine Alvarez, François Ferron

**Affiliations:** a Aix-Marseille Université and Laboratoire Architecture et Fonction des Macromolécules Biologiques (AFMB), CNRS – UMR-7257, 13288 Marseille, France; b European Virus Bioinformatics Center, Leutragraben 1, 07743 Jena, Germany

**Keywords:** *Arenaviridae*, exonucleases, Mopeia virus, Lymphocytic choriomeningitis virus, Lassa virus, alendronate, compound optimization, metal chelation

## Abstract

Arenaviruses are emerging enveloped negative-sense RNA viruses that cause neurological and hemorrhagic diseases in humans. Currently, no FDA-approved vaccine or therapeutic agent is available except for ribavirin, which must be administered early during infection for optimum efficacy. A hallmark of arenavirus infection is rapid and efficient immune suppression mediated by the exonuclease domain encoded by the nucleoprotein. This exonuclease is therefore an attractive target for the design of novel antiviral drugs since exonuclease inhibitors might not only have a direct effect on the enzyme but could also boost viral clearance through stimulation of the innate immune system of the host cell. Here, *in silico* screening and an enzymatic assay were used to identify a novel, specific but weak inhibitor of the arenavirus exonuclease, with IC_50_ values of 65.9 and 68.6 µ*M* for Mopeia virus and Lymphocytic choriomeningitis virus, respectively. This finding was further characterized using crystallographic and docking approaches. This study serves as a proof of concept and may have assigned a new therapeutic purpose for the bisphosphonate family, therefore paving the way for the development of inhibitors against *Arenaviridae*.

## Abstract 2

Virtual screening, molecular docking and molecular characterization of an *Arenaviridae* nucleoprotein exonuclease–inhibitor complex provided insight for structure-based drug design.

## 1. Introduction

The genus *Mammarenavirus* belongs to the *Arenaviridae*, a neglected family of enveloped negative-sense RNA viruses associated with neurological and hemorrhagic diseases in humans. In South America, Machupo, Guanarito, Junin, Sabia and Chapare viruses are responsible for hemorrhagic fever (Bowen *et al.*, 1997 ▸), while in Africa Lujo virus (Briese *et al.*, 2009 ▸) and Lassa virus (LASV) constitute major public health concerns (Mofolorunsho, 2016 ▸; Peterson *et al.*, 2014 ▸; Yun & Walker, 2012 ▸). LASV is by far the greatest public health concern as its geographical distribution overlaps with those of Ebola virus and other hemorrhagic viruses, which have very similar clinical symptoms. LASV accounts for an estimated 300 000–500 000 infections annually, leading to permanent and severe hearing loss and to death in the most severe cases (1% of the total number of infections; Günther & Lenz, 2004 ▸; Yun *et al.*, 2016 ▸). The case-fatality rate amongst patients in the 2016 outbreak was determined to be 37.9% with a spreading scenario (Nigeria, Benin, Togo, Liberia and imported cases in Germany and Sweden; World Health Organization, 2016*a* ▸,*b* ▸,*c* ▸, 2017*a* ▸,*b* ▸). The most recent outbreak in 2019 lasted two months in Nigeria, with a fatality rate amongst confirmed cases of 14% (World Health Organization, 2022 ▸). There is currently no FDA-licensed vaccine or therapeutic agent to protect against LASV infection except for ribavirin if administrated early in the infection.

Mammarenaviruses have a bisegmented genome composed of L (large; ∼7.3 kb) and S (small; ∼3.5 kb) segments (Buchmeier *et al.*, 2007 ▸). Both segments encode two proteins. The L segment encodes protein L, which is the main actor in transcription and replication through an endonuclease domain (Morin *et al.*, 2010 ▸) and the RNA-dependent RNA polymerase (RdRp) domain (Ferron *et al.*, 2017 ▸). The second protein of the L segment is the matrix RING-finger protein Z. The S segment encodes the precursor of the mature virion glycoprotein GP-C, which gives GP-1 and GP-2 after post-translational cleavage, and the nucleoprotein (NP). NP forms a polymer protecting the genomic (and anti-genomic) RNA (RNA_v_) and is involved in the suppression of innate immunity (Hastie, Kimberlin *et al.*, 2011 ▸; Jiang *et al.*, 2013 ▸; Qi *et al.*, 2010 ▸). L and NP, together with RNA_v_, form an active ribonucleic complex for replication and transcription that remains in the cytoplasm (Pinschewer *et al.*, 2003 ▸). One remarkable characteristic of arenavirus infection is the persistence of the infection (Rawls *et al.*, 1981 ▸). This is partly due to the ability of arenaviruses to very efficiently control the innate immune response of the infected host. NP and Z are directly involved in the regulation of type I interferons (IFN-α and IFN-β) and in the sequestration of interferon-stimulated genes (ISGs) (Meyer & Ly, 2016 ▸; Xing *et al.*, 2015 ▸). NP possesses an exonuclease domain that is involved in suppressing double-stranded RNAs (dsRNAs). The latter are markers of viral infection, which triggers the innate immunity response of the host. Recently, the role of the exonuclease domain was proposed to also extend to replication (Huang *et al.*, 2015 ▸; Yekwa *et al.*, 2019 ▸), promoting NP as a cornerstone protein in arenavirus infection. Due to its role in the suppression of innate immunity and potentially in replication, NP exo­nuclease (NP-exo) is an attractive target for the development of antiviral molecules.

NP-exo structures from Lymphocytic choriomeningitis virus (LCMV), LASV and Mopeia virus (MOPV) have previously been characterized in the presence of divalent ions (Mg^2+^ or Mn^2+^; Hastie, Kimberlin *et al.*, 2011 ▸; Jiang *et al.*, 2013 ▸; Yekwa *et al.*, 2017 ▸). The general mechanism for RNA hydrolysis follows a two-metal-ion mechanism as described by Steitz & Steitz (1993 ▸). It involves two metal ions positioned in the conserved catalytic pocket defined by the residues Asp390, Glu392, Asp467 and Asp534 (in MOPV) and across the target phosphodiester bond. In the previously reported structures (Yekwa *et al.*, 2017 ▸) only one metal ion was observed and the second metal ion was transiently observed in the presence of RNA substrate (Jiang *et al.*, 2013 ▸). The domain is specifically active against dsRNA in the presence of Mg^2+^ and this activity is boosted in the presence of Mn^2+^.

In this study, we present the identification of a compound that could be considered to be a hit compound in the development of antivirals against *Arenaviridae* 3′–5′ exonuclease activity. We used an *in silico* approach including the virtual screening of a library of sorted molecules to identify a compound that inhibits the activities of MOPV and LCMV NP-exo *in vitro*. We further consolidated this finding with crystallo­graphic and docking studies using MOPV NP-exo.

## 2. Materials and methods

### 2.1. Compound library

To provide valuable starting points for open virtual screening, we used a diverse and drug-like chemical library (MTiOpenScreen Diverse-lib) containing 99 288 molecules (Labbé *et al.*, 2015 ▸). A subset of 10 000 ligands were screened in this study using lead-like pre-filter selection by flexible physico-chemical criteria.

### 2.2. Structure-based virtual screening

We used the structure of the C-terminal exonuclease domain of the Mopeia virus nucleoprotein (MOPV NP-exo) which was solved in complex with an Mg^2+^ ion (PDB entry 5lrp; Yekwa *et al.*, 2017 ▸). An initial virtual screening was performed using 10 000 ligands from the MTiOpenScreen Diverse-lib using the web server (https://bioserv.rpbs.univ-paris-diderot.fr/services/MTiOpenScreen/). We provided the following input information: (i) a PDB file of the protein structure and (ii) the center and grid dimensions (*a* = *b* = *c* = 30 Å) of the active site of the protein. The uploaded PDB file was automatically cleaned by removing all HETATMS and hydrogen bonds using *MGL Tools* (Labbé *et al.*, 2015 ▸). This screen generated a list of the top-ranked predicted binding poses and energies of 100 ligands.

### 2.3. Molecular docking

The 3D structure of MOPV NP-exo (PDB entry 5lrp) was energy-minimized by the steepest-gradient method of energy minimization followed by conjugate-gradient minimization, using the *MMTK* and *Amber* packages (Cornell *et al.*, 1995 ▸; Hinsen, 2000 ▸; Lindorff-Larsen *et al.*, 2010 ▸). Mol2 and PDB file formats of the ligands and receptor were converted to PDBQT format using *UCSF Chimera* (Pettersen *et al.*, 2004 ▸) prior to docking. All water and solvent atoms of the protein were removed and polar hydrogens and polar charges were added onto the ions and ligand prior to docking. The protein was kept rigid, while the ligand was allowed to rotate and explore more flexible binding modes. Docking of the ligands onto MOPV NP-exo was performed iteratively using *AutoDock Vina* version 1.1.2 (Trott & Olson, 2010 ▸). The best poses from the first round of docking were used as seeds for the second round. The grid box size was further optimized to dimensions of *a* = 23.2, *b* = 25.6, *c* = 21.2 Å, thus covering the binding pocket. The default scoring function was used for docking. Binding modes of the docked complexes were obtained and sorted based on their binding energies. Ions and amino-acid residues present at a distance of less than 3 Å were considered to be binding partners of the ligands.

Geosmin synthase from *Streptomyces coelicolor* (ScGS) in complex with three Mg^2+^ ions and alendronate (ALD; PDB entry 5dz2; Harris *et al.*, 2015 ▸) was used as a control experiment. The same protocol was applied to the 3D structure of ScGS depleted of solvent and ALD. The ScGS–ALD binding pose resulting from our docking protocol was superimposed onto the experimentally derived structure. The interaction figures representing the docked complexes were generated with *UCSF Chimera*.

Exploration of various possible dynamic poses of ALD in the MOPV NP-exo structure with Mn^2+^ obtained in this study (PDB entry 6sx8) was performed with *AutoDock Vina* using a grid (of dimensions *a* = 29.34, *b* = 28.71, *c* = 28.00 Å) with the ion placed in the center of the grid to allow an extensive exploration of possible poses.

### 2.4. Cloning, gene expression and purification of proteins

The MOPV NP-exo (residues 365–570; UniProt P19239) and LCMV NP-exo (residues 357–559; NCBI RefSeq NP_694852) genes were cloned by recombination (Gateway, Invitrogen) into the pETG20A expression vector, which adds a cleavable N-terminal thioredoxin–hexahistidine tag. The accuracy of the DNA construct sequence was verified by sequencing.

The vectors were used to transform T7 Express competent *Escherichia coli* cells (New England Biolabs) carrying the pRARE plasmid (Novagen). The bacteria were cultured in Turbo Broth medium (AthenaES) at 310 K. When the OD_600 nm_ reached 0.6, gene expression was induced with 0.5 m*M* isopropyl β-d-1-thiogalactopyranoside (IPTG) and the cells were incubated overnight at 290 K while agitating at 250 rev min^−1^ in the presence of 100 µ*M* ZnCl_2_. The bacteria were pelleted by centrifugation and stored at 193 K.

The cell pellets were resuspended in lysis buffer (20 m*M* HEPES pH 7.5, 300 m*M* NaCl, 5 m*M* imidazole, 5% glycerol, 0.25 mg ml^−1^ lysozyme, 100 µg ml^−1^ DNase, 0.1 m*M* phenylmethylsulfonyl fluoride, 0.1% Triton X-100) at 193 K, followed by sonication. The lysate was then cleared by centrifugation at 20 000*g* at 277 K for 30 min. Each protein was first purified by immobilized metal-affinity chromatography (IMAC) using 5 ml HisPur cobalt resin (Thermo Scientific). The His-tagged proteins were recovered using elution buffer consisting of 20 m*M* HEPES pH 7.5 containing 200 m*M* NaCl, 250 m*M* imidazole and 5% glycerol. The tag was cleaved by TEV protease followed by a second IMAC step to remove non­cleaved protein and His-tagged TEV protease. The final step consisted of gel filtration using a Superdex 200 column (Cytiva) in 10 m*M* HEPES pH 7.5, 150 m*M* NaCl, 3 m*M* MnCl_2_, 5% glycerol. The quality of both proteins was verified by SDS–PAGE and mass spectrometry.

The gene for human t3′ repair exonuclease 2 (hTREX2) cloned into the pLM-303 vector was kindly provided by Thomas Hollis, Wake Forest University Health Sciences, USA. This construct consists of hTREX2 as a fusion protein with maltose-binding protein (MBP). The hTREX2-MBP fusion was expressed in *E. coli* BL21 (DE3) pLysS cells (Novagen). The transformed cells were cultured in Luria Broth medium (AthenaES) at 310 K in the presence of 50 mg ml^−1^ kanamycin and 34 mg ml^−1^ chloramphenicol. When the OD_600 nm_ reached 0.4–0.6, protein expression was induced with 1 m*M* IPTG and was completed overnight at 290 K. The cells were collected by centrifugation. The cell pellets were lysed by sonication in 100 m*M* Tris pH 7.5 containing 300 m*M* NaCl, 5 m*M* imidazole, 5% glycerol, 0.25 mg ml^−1^ lysozyme, 50 µg ml^−1^ DNase, 0.1% Triton X-100 and 0.1 m*M* EDTA at 193 K. The lysate was clarified by centrifugation at 20 000*g* and 277 K for 30 min. The supernatant was harvested and loaded onto an affinity-chromatography column containing 5 ml amylose resin (GE Biosciences). The protein was eluted with 50 m*M* Tris pH 7.5, 200 m*M* NaCl, 5 m*M* imidazole, 5% glycerol, 0.1 m*M* EDTA, 1 m*M* DTT, 10 m*M* maltose. The MBP was removed by treatment with PreScission Protease (GE Healthcare) at 277 K for 20 h while being dialyzed in buffer consisting of 50 m*M* Tris pH 7.5, 150 m*M* NaCl, 5% glycerol, 0.1 m*M* EDTA, 2 m*M* DTT. hTREX2 was separated from cleaved MBP using an ion-exchange column (HiTrap QFF 5 ml, Cytiva) and finally purified using a size-exclusion column (HiLoad 16/60 Superdex 75, Cytiva) in 20 m*M* Tris pH 7.5, 150 m*M* NaCl, 5% glycerol, 2 m*M* DTT. hTREX2 was concentrated to 5 mg ml^−1^ and stored at 253 K until use.

### 2.5. Enzymatic activity assays

#### 2.5.1. RNA substrate

The purified (HPLC) synthetic HP4 RNA (Biomers) used as a substrate for *in vitro* assays is a stable RNA hairpin (5′-UGACGGCCCGGAAAACCGGGCC-3′, Δ*G* = −14.8 kcal mol^−1^). The RNA was labeled at its 5′ end with [γ-^32^P]-ATP (Perkin–Elmer) using T4 polynucleotide kinase (New England Biolabs) according to the manufacturer’s instructions.

#### 2.5.2. DNA substrates

HPLC-grade synthetic ssDNA (20 nt, 5′-ACTGGACAAATACTCCGAGG-3′) and Y-shaped DNA (24 nt, 5′-TTAAGGCCCTCTTTAGGGCCAAGG-3′) from Eurofins Genomics were used as substrates for the *in vitro* hTREX2 assays. The DNA substrates were labeled with [γ-^32^P]-ATP using T4 polynucleotide kinase (New England BioLabs) following the manufacturer’s instructions.

#### 2.5.3. Inhibitors

Alendronate (ALD) sodium trihydrate (C_4_H_12_NaNO_7_P_2_·3H_2_O) was purchased from Sigma.

#### 2.5.4. Exonuclease activity assay

The activity of MOPV NP-exo was measured using 0.25 µ*M* NP-exo in a reaction buffer consisting of 20 m*M* Tris pH 8.0 containing 5 m*M* MnCl_2_ (or MgCl_2_), 2 m*M* DTT, 0.1 m*M* EDTA and 1.5 µ*M* radiolabeled HP4 RNA substrate. The negative control consisted of the same setup without enzyme. The inhibition assays were carried out in the presence of a given concentration of ALD. NP-exo, ions and inhibitor were incubated in reaction buffer at room temperature for 5 min and the reaction was then triggered by adding radiolabeled HP4 RNA substrate followed by incubation at 310 K. The reaction was stopped after different time intervals (0, 5 and 30 min) by adding formamide gel-loading buffer (FBD; 95% deionized formamide, 0.1% bromophenol blue, 0.1% xylene cyanol FF, 10 m*M* EDTA) in a 2:3(*v*:*v*) ratio (sample:FBD buffer). The products were heated at 343 K for 10 min and rapidly cooled on ice before being analyzed by denaturing PAGE using sequencing gels [20% acrylamide:bisacrylamide (19:1), 8 *M* urea with TTE buffer (89 m*M* Tris pH 8.0, 28 m*M* taurine (2-aminoethanesulfonic acid), 0.5 m*M* EDTA]. The RNA product bands were visualized using photo-stimulated plates and an Amersham Typhoon biomolecular imager (Cytiva). RNA degradation was quantified using *ImageQuant TL* (Cytiva). IC_50_ values were determined using *GraphPad Prism* version 7.0. Experiments were performed in triplicate.

hTREX2 assays were carried out with 0.25 µ*M* hTREX2 in reaction buffer consisting of 20 m*M* Tris pH 7.0, 120 m*M* NaCl pH 7.0, 5 m*M* MnCl_2_ (or MgCl_2_) and 0.5 µ*M* radiolabeled DNA substrates. The negative controls were carried out in the absence of enzyme. An inhibition assay was performed in the presence of 100 µ*M* ALD. The reactions were started by adding DNA substrates and were incubated for 30 min at 310 K. The reactions were stopped by adding 1.5 volumes of FBD buffer and the samples were analyzed as described above. Experiments were carried out in duplicate.

### 2.6. Protein–ligand crystallization and structure characterization

#### 2.6.1. Crystallization

The protein was concentrated to 15 mg ml^−1^. Screening was performed in a SWISSCI 96-well plate using The PEGs Suite and the sitting-drop vapor-diffusion method. Three different volumes of protein (300, 200 and 100 nl) were mixed with a constant volume of precipitant (100 nl) using a nanodrop dispensing robot (Mosquito, TTP Labtech). The crystallization plate was kept at 277 K overnight and was then changed to 291 K. The optimal crystallization conditions for MOPV NP-exo complexed with Mn^2+^ were obtained by mixing 200 or 100 nl protein solution with 100 nl reservoir solution consisting of 0.1 *M* MES pH 6.2–6.8, 24–26%(*m*/*w*) PEG 8000. Once the condition with Mn^2+^ had been established, crystals were soaked in several concentrations of MnCl_2_ (from 1 to 2 m*M*) and ALD (from 1 to 4 m*M*) for times ranging from 5 min to 24 h. The crystals were protected with a cryoprotectant consisting of 20% glycerol and 80% reservoir solution and flash-cooled in liquid nitrogen at 100 K.

#### 2.6.2. Data collection and structure

Diffraction data were collected on the PROXIMA-1 beamline at Synchrotron SOLEIL, Saint-Aubin, France using the large in-vacuum PILATUS 6M detector (Dectris, Switzerland) at a wavelength of 0.97857 Å with 0.1° oscillation and 0.097 s exposure time and also on the ID23-1 beamline at the European Synchrotron Radiation Facility (ESRF), Grenoble, France using the large in-vacuum PILATUS 6M-F detector (Dectris, Switzerland) at a wavelength of 0.9787 Å with 0.1° oscillation, 0.037 s exposure time and a temperature of 100 K. The collected data were subsequently processed using the *autoPROC* toolbox (Von­rhein *et al.*, 2011 ▸). The data were integrated using *XDS* (Kabsch, 2010 ▸), analyzed with *POINTLESS* (Evans, 2006 ▸) and scaled with *AIMLESS* (Evans & Murshudov, 2013 ▸). The structure was solved using MOPV NP-exo (PDB entry 5ls4; Yekwa *et al.*, 2017 ▸) as a search model. The search model was prepared by removing all ligands, metal ions and water molecules from the original structure. Phases were obtained by molecular replacement using *Phaser* (McCoy *et al.*, 2007 ▸). Refinement was performed by successive and alternating rounds of refinenent with *BUSTER* (Bricogne *et al.*, 2018 ▸) or *Phenix* (Liebschner *et al.*, 2019 ▸) and model improvement using *Coot* (Emsley *et al.*, 2010 ▸). The structures were validated using *MolProbity* (Williams *et al.*, 2018 ▸) and *PROCHECK* (Laskowski *et al.*, 1993 ▸). Structural analysis was performed using *UCSF Chimera* (Pettersen *et al.*, 2004 ▸). The X-ray structure was validated and deposited in the PDB. Data-collection, refinement statistics and accession numbers are shown in Table 1 ▸.

## 3. Results

### 3.1. Structure-based virtual screening and molecular docking

**Figure 1:**
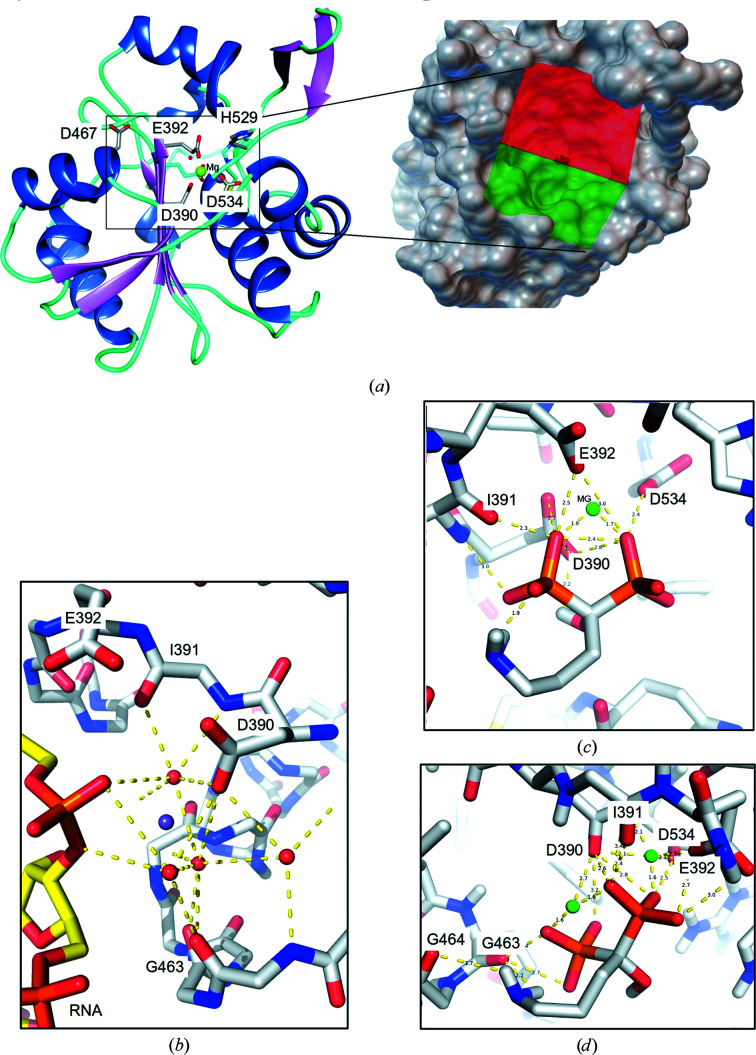
Screening and docking experiments against MOPV NP-exo. (*a*) The crystal structure of MOPV NP-exo used for docking studies (PDB entry 5lrp) presented as a ribbon diagram of the protein with helices in blue, β-strands in pink and loops in cyan. Active-site residues are shown with their side chains as sticks (C atoms in gray, O atoms in red and N atoms in blue). The coordination of the Mg^2+^ ion (green sphere) is also illustrated. The expanded square corresponds to the red/green box shown on the molecular-surface representation of the MOPV NP-exo structure. It represents the grid box used to define the docking space covering the entire active site. (*b*) Interactions of RNA with the catalytic site of LASV NP-exo and a single Mn^2+^ ion (purple sphere; PDB entry 4gv6) for reference. (*c*) Interactions of ALD inside the MOPV NP-exo active site in the presence of a single Mg^2+^ ion obtained by *AutoDock Vina*. (*d*) Interaction of ALD with the MOPV NP-exo model containing two Mg^2+^ ions after *AutoDock Vina*.

The high-resolution (1.9 Å) crystal structure of MOPV NP-exo complexed with Mg^2+^ (PDB entry 5lrp) was used for this study. The active site comprises five catalytic residues, Asp390, Glu392, Asp534, Asp467 and His529, which form an acidic cavity. Three of these residues directly coordinate one of the catalytic ions, in this case Mg^2+^, as shown in Fig. 1 ▸(*a*). In order to identify ligands with a high affinity for MOPV NP-exo, with the aim of inhibiting its 3′–5′ exonuclease activity, we performed a structure-based virtual screen of a total of 10 000 ligands from the MTiOpenScreen Diverse-lib (see Section 2). This diverse library is composed from the sampling of 12 chemical libraries from the PubChem BioAssay Database containing a drug-like compound collection. Our initial structure-based virtual screening using the MTiOpenScreen server generated a total of 100 ligands that could interact with MOPV NP-exo at its active site. The ligands were ranked according to their predicted binding energies, with a lowest predicted binding energy of −9.44 kcal mol^−1^. Supplementary Tables S1 and S2 show the predicted binding energies and physicochemical properties as well as the structural formulae of the top ten hits.

Manual inspection of the list of the top ten ligand hits allowed us to identify the presence of a geminal bisphos­phonate molecule, alendronate (ALD), which mimics the phosphate of RNA substrates in its pose, as shown in Figs. 1 ▸(*b*) and 1 ▸(*c*). Structurally, ALD is a chemically stable derivative of inorganic pyrophosphate (PP_i_).

Using *AutoDock Vina*, we redocked ALD with one or two Mg^2+^ ions into the catalytic site of MOPV NP-exo. Since there is no crystal structure of an arenavirus NP-exo domain in complex with ALD to the best of our knowledge, in order to access the efficiency of prediction of our model we validated the docking protocol using the structure of geosmin synthase from *S. coelicolor* (ScGS) complexed with three Mg^2+^ ions and ALD [Supplementary Figs. S1(*a*) and S1(*b*)]. We obtained two models [Supplementary Figs. S1(*c*), S1(*d*) and S1(*e*)] with an overall root-mean-square deviation (r.m.s.d.) between the two ALD molecules of between 1.68 and 2.07 Å, but with an r.m.s.d. of between 0.68 and 1.28 Å at the phosphonate level [Supplementary Fig. S1(*f*)]. We concluded that our docking protocol can predict conformations that are in agreement with experimentally determined structures.

The best ALD binding energy obtained after *AutoDock Vina* was −7.62 kcal mol^−1^, which is consistent with the result from screening (−7.6 kcal mol^−1^; Supplementary Table S2). The binding mode of ALD to MOPV NP-exo and its interactions with the Mg^2+^ ion in site *A* (present in PDB entry 5lrp), as well as the active-site residues, is shown in Fig. 1 ▸(*c*). The structure of ALD contains an alkyl chain of five C atoms substituted by an hydroxyl group and terminated by a primary amine and two negatively charged geminal phosphonate groups PO(O^−^)_2_ that are able to establish electrostatic interactions. The phosphonate groups establish hydrogen-bond interactions with Asp534 (2.4 Å), Asp390 (2.7 Å) and Ile391 (3.0 Å) and direct polar interactions with Glu392 (2.5 and 3.0 Å) and the Mg^2+^ ion (1.7 and 1.9 Å). As the active site of *Arenaviridae* NP-exo can harbor two catalytic metal ions, a model of MOPV NP-exo with two Mg^2+^ ions was generated based on the homologous structure of a dsRNA bound to LASV NP-exo containing two Mn^2+^ ions (PDB entry 4gv9; Jiang *et al.*, 2013 ▸). The dsRNA from the original crystal structure was removed, while conserving the second Mg^2+^ ion within the MOPV model that was then used for docking. As Fig. 1 ▸(*d*) shows, in the presence of two Mg^2+^ ions ALD positions itself in such a way that the two phosphonate groups establish polar interactions with both metal ions. In addition to the residues cited earlier, it also makes polar interactions with the main chains of Gly463 and Gly464. The binding energy of the best ALD pose in the MOPV NP-exo model with two metal ions (−16.7 kcal mol^−1^) is approximately two times lower compared with that of the best pose within the model containing a single metal ion (−7.62 kcal mol^−1^).

### 3.2. Alendronate inhibits 3′–5′ exo­nuclease activity of MOPV NP-exo and LCMV NP-exo in the presence of Mn^2+^ ions

**Figure 2:**
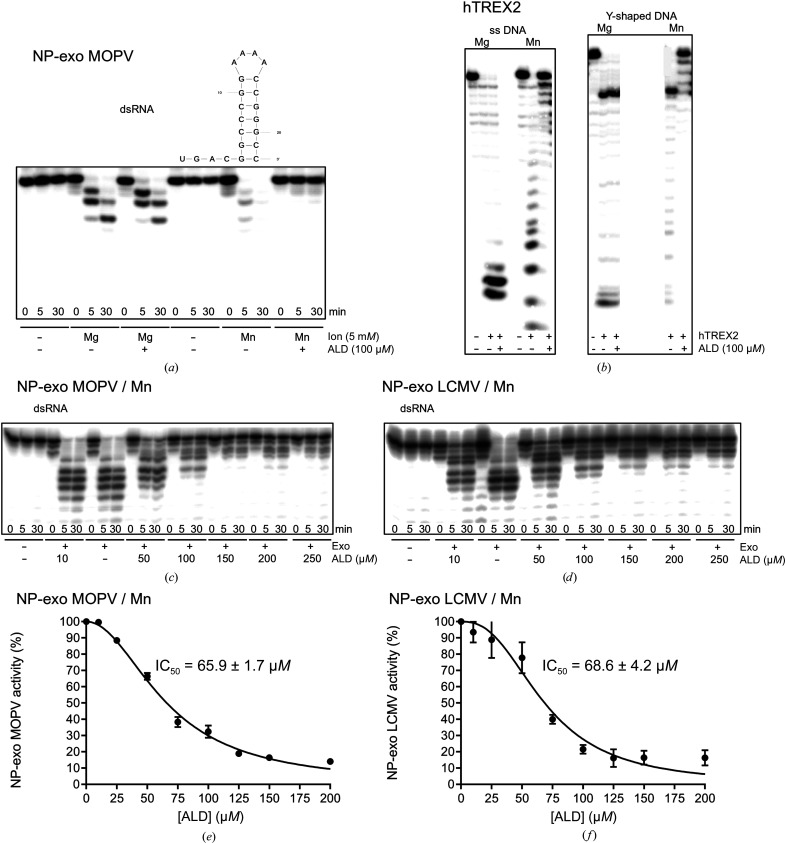
ALD inhibition of 3′–5′ exonuclease activity. (*a*) MOPV NP-exo was incubated with HP4 dsRNA in the absence or presence of the catalytic ions Mg^2+^ or Mn^2+^ and a fixed concentration of ALD (100 µ*M*). Reaction samples were taken after time intervals of 0, 5 and 30 min. The products were analyzed on urea–PAGE and visualized by autoradiography. (*b*) Inhibition of hTREX2 activity in the presence of 100 µ*M* ALD. Left, inhibition of hTREX2 activity on single-stranded DNA (ssDNA); right, inhibition of hTREX2 activity using Y-shaped DNA with four-nucleotide 3′- and 5′-overhangs. (*c*) and (*d*) show representative gels showing the effect of ALD on the exonuclease activity of MOPV NP-exo and LCMV NP-exo, respectively. (*e*) and (*f*) are dose–response curves, from which IC_50_ values were calculated. For IC_50_ determination, the percentage of activity was measured by quantifying the total product formed after 5 min. IC_50_ values were then determined by fitting the dose–response curves. Reactions were performed in triplicate.

As ALD is readily available commercially, we decided to evaluate its ability to inhibit 3′–5′ exonuclease activity. The method is based on the ability of the enzyme to hydrolyze the 22-nucleotide hairpin HP4 RNA that has been radiolabeled at its 5′-end and whose 3′-end is engaged in the hairpin [dsRNA; Fig. 2 ▸(*a*)]. The effect of 100 µ*M* ALD in the presence of 5 m*M* Mg^2+^ or Mn^2+^ as the catalytic ion was tested with MOPV NP-exo. Fig. 2 ▸(*a*) shows that HP4 dsRNA is degraded in the presence of both metal ions. The substrate band disappears after 5 min and degradation proceeds further up to 30 min. ALD does not inhibit dsRNA hydrolysis in the presence of Mg^2+^ but inhibits degradation completely in the presence of Mn^2+^. Interestingly, the same Mn^2+^-ion-dependent inhibition was observed when ssDNA and Y-shaped DNA were degraded by recombinant hTREX2 in the presence of Mg^2+^ or Mn^2+^ [Fig. 2 ▸(*b*)]. Here, we also observed inhibition by 100 µ*M* ALD in the presence of Mn^2+^.

Subsequently, MOPV NP-exo or LCMV NP-exo and HP4 dsRNA were incubated with increasing concentrations of ALD (10–250 µ*M*) in the presence of Mn^2+^. Figs. 2 ▸(*c*) and 2 ▸(*d*) show that both enzymes are inhibited and that inhibition is almost complete at 150 µ*M* ALD. In order to determine IC_50_ values, both reactions were then performed in triplicate in the presence of 10, 25, 50, 75, 100, 125, 150 and 200 µ*M* ALD. Quantitative comparison of the disappearance of the substrate band after 5 min incubation in comparison to the absence of ALD was used to calculate the percentage of activity at a certain ALD concentration [Figs. 2 ▸(*e*) and 2 ▸(*f*)]. IC_50_ values were determined by *GraphPad Prism* to be 65.9 ± 1.7 µ*M* ALD and 68.6 ± 4.2 µ*M* ALD for MOPV NP-exo and LCMV NP-exo, respectively.

In conclusion, although we expected from our screening and docking approaches that ALD could inhibit arenavirus NP-exo activity in the presence of Mg^2+^ ions, we obtained Mn^2+^-specific inhibition with moderate IC_50_ values.

### 3.3. Crystal structures of MOPV NP-exo complexed with Mn and soaked with ALD

**Figure 3:**
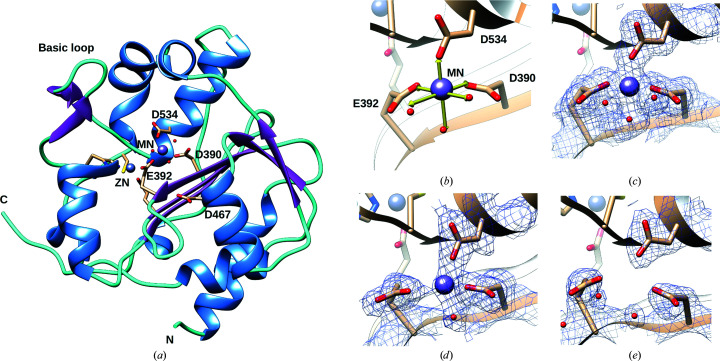
Crystal structure of MOPV NP-exo complexed with Mn^2+^ and soaked in the presence of ALD. (*a*) Ribbon representation of MOPV NP-exo complexed with Mn^2+^ (PDB entry 6sx8). Mn^2+^ is represented as a purple sphere and coordinated by three water molecules (red spheres). The protein structure color code follows that in Fig. 1 ▸. Catalytic residues are labeled. Three of the residues coordinating the structural Zn^2+^ ion (gray sphere) are also shown, with their side chains in stick representation. (*b*) Enlargement of the Mn^2+^ ion in the active site of MOPV NP-exo complexed with Mn^2+^. The theoretical Mn^2+^ coordination by the catalytic residues and water molecules is illustrated by yellow arrows. (*c,* *d*, *e*) The same view as in (*b*) presenting a 2*F*_o_ − *F*_c_ map corresponding to the metal ion-binding site contoured at 1.3σ at different soaking times: (*c*) 6 min (PDB entry 6t6l), (*d*) 12 min (PDB entry 6t2a) and (*e*) overnight (PDB entry 6sy8).

In order to structurally investigate the mechanism of action of ALD in inhibiting 3′–5′ exonuclease activity in the presence of Mn^2+^ ions, and to determine whether ALD could fix the second catalytic ion, we crystallized MOPV NP-exo in complex with Mn^2+^ and soaked the crystals with ALD using different soaking times (6 min to overnight). We obtained four structures, which are listed in Table 1 ▸. The first structure was complexed with one Mn^2+^ ion and represents our reference structure [PDB entry 6sx8; Fig. 3 ▸(*a*)]. Its general structure and the location of the ion are similar to those reported with Mg^2+^ and Ca^2+^ (Yekwa *et al.*, 2017 ▸). MOPV NP-exo in complex with Mn^2+^ consists of six mixed strands forming a central β-sheet sandwiched by three α-helices on one side and five α-helices on the opposite side. Two flexible regions are present within the structure. Two antiparallel strands form a ‘basic loop’ (residues 514–526) near the catalytic pocket, and the C-terminal arm (residues 549–570) extends away from the protein core. The MOPV NP-exo structure shows a structurally conserved zinc-binding domain. The Zn^2+^ ion is coordinated by Glu400, Cys507, His510 and Cys530. The Mn^2+^ ion is coordinated by the three catalytic residues Asp390, Glu392, Asp534 and three H_2_O molecules [Fig. 3 ▸(*b*)]. The presence of Mn^2+^ and Zn^2+^ ions in the crystal was confirmed by an X-ray fluorescence scan (Supplementary Fig. S2). The other three structures were obtained after soaking crystals with ALD for 6 min (PDB entry 6t6l), 12 min (PDB entry 6t2a) and overnight (PDB entry 6sy8). The overall structures are not changed by the soaking except for the fact that the basic loop is clearly defined in all three structures obtained from soaking with ALD, in contrast to that complexed with Mn^2+^, indicating that ALD contributes to lowering the energy of this structural element. The structure of the zinc-binding domain and the four residues forming the conserved zinc-binding site are not affected by soaking with ALD. In the active site, the densities around the water molecules and the ion are significantly weakened, indicating a gradual depletion of the ion in the structure. The three water molecules are still present after 6 min of soaking [Fig. 3 ▸(*c*)] but start to disappear after 12 min [Fig. 3 ▸(*d*)]. The Mn^2+^ ion is still present in the structures, but its occupancy was refined at 52% and 34%, respectively, to reflect its disappearance. This is indicative that there is a depletion of the Mn^2+^ in the structure in the presence of ALD. Finally, after 40 min (not shown) or overnight soaking [Fig. 3 ▸(*e*)] we observed no density in the catalytic site, reflecting the complete disappearance of the Mn^2+^ ion. During refinement we observed positive density around the Mn^2+^ ion that was larger than a regular water molecule [Supplementary Fig. S3(*a*)], but we were not able to refine it with an ALD molecule, indicating that the process of the binding of ALD to MOPV NP-exo complexed to Mn^2+^ is extremely dynamic and that ALD is not stabilized by strong interactions with the protein.

**Figure 4:**
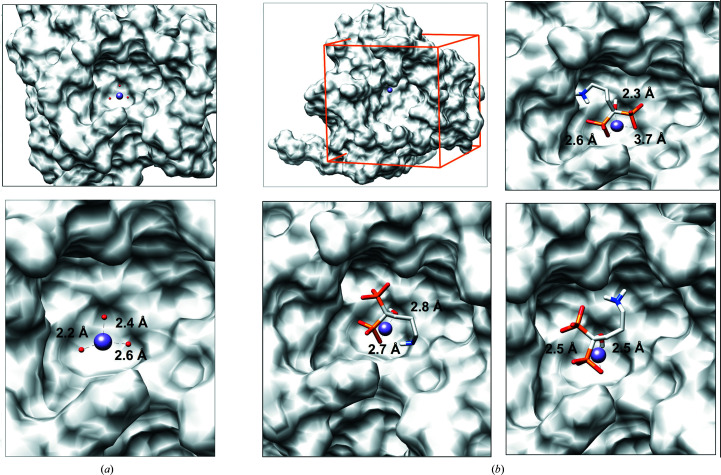
Exploration of potential ALD poses resulting from docking experiments using the crystal structure of MOPV NP-exo complexed with Mn^2+^. (*a*) Molecular-surface representation of the MOPV NP-exo structure complexed with Mn^2+^, which is represented as a purple sphere and coordinated by three water molecules (red spheres). The positioning of the water molecules serves as a reference for potential binding modes. Their distances from the ion are given in the enlargement (lower panel). (*b*) Molecular-surface representation of the MOPV NP-exo structure complexed with Mn^2+^ depleted of water molecules. The orange box corresponds to the relaxed docking grid encompassing the active site; the ion is at its center. The three best poses compatible with the observed unrefined density (Supplementary Fig. S3) and the positions of the water molecules are shown. The given distances are compatible with the observed distances to coordinated waters.

Using our structural data we redocked ALD, taking into consideration the position of the Mn^2+^ ion, the coordinated water molecules [Fig. 4 ▸(*a*)] and a positive blob of unrefined density observed in the structure after soaking with ALD for 6 min [Supplementary Fig. S3(*a*)]. After applying the *AutoDock Vina* protocol we obtained a series of possible ALD poses [Supplementary Fig. S3(*b*), left]. Molecules for which at least two O atoms of ALD overlap with the observed position of a water molecule and that are compatible with the observed unrefined density led to the selection of three poses [Fig. 4 ▸(*b*) and Supplementary Fig. S3(*b*), right]. These poses allow the proposal that the O atoms of the ALD phosphonates displace the coordinated water molecules; by doing so they weaken the affinity of the ion towards the protein, allowing its sequestration by ALD.

In conclusion, our structure–function analysis of MOPV NP-exo with Mn^2+^ and ALD indicate that the inhibitory effect of ALD is specific to Mn^2+^ through specific metal chelation.

## 4. Discussion

Several pieces of evidence have demonstrated that metal-dependent enzymes are important targets for antiviral discovery (Carcelli *et al.*, 2017 ▸; DiScipio *et al.*, 2022 ▸). The chelation of metal-ion cofactors is therefore an interesting and approved strategy for the development of novel antiviral therapies (Rogolino *et al.*, 2012 ▸). The arenavirus 3′–5′ exo­nuclease is a divalent metal-dependent enzyme that requires Mn^2+^ (or Mg^2+^) as a cofactor. This enzyme degrades immunogenic dsRNA, a process thought to be used by arenaviruses to suppress type I interferon induction (Hastie, Liu *et al.*, 2011 ▸; Jiang *et al.*, 2013 ▸; Qi *et al.*, 2010 ▸). It has also been suggested that this 3′–5′ exonuclease might play an important role during RNA replication (Huang *et al.*, 2015 ▸). Moreover, the catalytic tetrad DEDD is completely conserved among the arenaviral enzymes (Hastie, Kimberlin *et al.*, 2011 ▸), thus making this enzyme an interesting target for the development of broad-spectrum antivirals across the *Arenaviridae* family.

Here, we used a structure-based virtual screening and molecular-docking approach to identify a ligand that inhibits the 3′–5′ exonuclease activity of two arenavirus NP-exo domains in an Mn^2+^-specific manner *in vitro*. Among the ten best ligands from the screening, we chose one compound based on its ability to bind through ion interaction at the active site, thus preventing binding of the phosphate of the RNA substrate of the enzyme. This choice was driven by our knowledge of the targeted active site, the mechanism of RNA hydrolysis by the arenavirus 3′–5′ exonuclease and the availability of a co-crystal structure of a dsRNA bound to LASV NP-exo (Yekwa *et al.*, 2019 ▸; Jiang *et al.*, 2013 ▸).

We soaked the compound to co-crystallize it with the NP-exo domain. Contrary to our expectations, ALD did not mimic the working substrate condition since it failed to retain a second ion. The complex with ALD did not form, but we observed depletion of the ion in the catalytic site over time. These results allow the proposal that ALD does not pre-bind an ion prior to entry to the catalytic site. We know from the structure of geosmin synthase that ALD is able to interact with up to three metal ions at once. In the case of MOPV NP-exo we observe a more dynamic mechanism. In our simulation, the phosphonate groups of ALD seem to establish polar interactions specifically with the catalytic Mn^2+^ ion in place of the water molecules. These interactions are in agreement with those seen between the negatively charged phosphate of the RNA substrate and the Mn^2+^ ion in the co-crystal structure of dsRNA bound to LASV NP-exo (Jiang *et al.*, 2013 ▸). From the structural study, ALD inhibits 3′–5′ exo­nuclease activity through metal-ion chelation but not through steric hindrance or competition with the substrate for the active site.

According to our *in vitro* analysis, ALD inhibits the activity of MOPV NP-exo and LCMV NP-exo with values in the micromolar range. Interestingly, it has no effect in the presence of Mg^2+^, but only inhibits the enzyme in the presence of Mn^2+^. It is not the first time that such discrimination of a compound towards metal ions has been observed in nucleases (Saez-Ayala *et al.*, 2019 ▸), and this reinforces our consideration that ALD inhibits 3′–5′ exonuclease activity through specific ion chelation. Control experiments using hTREX2 show a similar ion-dependency effect on two different types of DNA substrate. It was previously demonstrated that a few viral nucleases, such as that from influenza virus, use Mn^2+^ (Crépin *et al.*, 2010 ▸), while cellular enzymes such as hTREX2 are more prone to use Mg^2+^ (de Silva *et al.*, 2009 ▸). In this context, it would be possible to consider a limited effect on hTREX2 *in vivo* even at high concentrations of ALD. This molecule belongs to the bisphosphonate family (BP). Bisphosphonates are pyrophos­phate analogs and are inhibitors of the mevalonate pathway, mainly targeting farnesyl pyrophosphate synthase (FDPS; Rondeau *et al.*, 2006 ▸) and thus preventing prenylation of the GTPases Ras, Rho and Rab, which are key signaling proteins (Koshimune *et al.*, 2007 ▸; Rogers *et al.*, 2011 ▸). Moreover, BPs are inhibitors of bone resorption (Fleisch, 1991 ▸). BPs are also used to treat skeletal complications (Goffinet *et al.*, 2006 ▸; Van Acker *et al.*, 2016 ▸). Nitrogen-containing bisphosphonates (such as ALD) have been used for the treatment of osteoporosis and tumor-related hypercalcemia (Reszka & Rodan, 2004 ▸). There is increasing evidence that BPs can inhibit proliferation and induce apoptosis in a variety of human tumor cells such as myeloma, breast, pancreas and prostate cancer cells under various conditions (Senaratne *et al.*, 2000 ▸; Takahashi *et al.*, 2001 ▸; Virtanen *et al.*, 2002 ▸). To our knowledge, there has been no previous report of the use of BP in antiviral therapy. Other pyrophosphate analogs such as phosphonoformic acid (PFA, foscarnet) and phosphonoacetic acid (PAA) are known to be potent inhibitors of Herpes virus DNA polymerases (Eriksson *et al.*, 1982 ▸). Foscarnet is a broad-spectrum antiviral that is used for the treatment of human cytomegalovirus and also inhibits HIV reverse transcriptase (Marchand *et al.*, 2007 ▸). It inhibits DNA polymerases by trapping the polymerase–DNA complex in its untranslocated form. A structural study on a chimeric DNA polymerase complexed with foscarnet revealed its mode of binding within the active site, showing that foscarnet chelates metal ions while interacting with conserved residues of the finger domain that are critical for catalysis (Zahn *et al.*, 2011 ▸). Even if ALD is not a potent viral inhibitor this study shows that it is a moderate inhibitor *in vitro*, and it is proposed that pyrophosphate analogs might constitute promising lead molecules for the development of antivirals against the arenavirus exonuclease. Bisphosphonates are known to be very stable compounds in biological fluids due to their chemical structure, which makes them resistant to enzymatic hydrolysis by pyrophosphatases (for example acid phosphatase and alkaline phosphatase; Russell, 2006 ▸).

In a strategy against *Arenaviridae*, the idea of having a molecule that targets the viral nuclease to restore the innate immune response, even partially, is an interesting lead to follow (Saez-Ayala *et al.*, 2019 ▸). It would break the persistence cycle of infection, allowing the cells to have a better chance of defense. It would also be interesting to consider this type of molecule in combination with other potential candidates that target other aspects of the viral life cycle, such as the replication inhibitor T-705 (Mendenhall, Russell, Smee *et al.*, 2011 ▸; Mendenhall, Russell, Juelich *et al.*, 2011 ▸).

## 5. Conclusions

We report four crystallographic structures of MOPV NP-exo: one in complex with an Mn^2+^ ion and three showing its disappearance over time in the presence of ALD. We have demonstrated the efficacy of ALD as an *in vitro* inhibitor of NP-exo from MOPV and LCMV, which are two model viruses for the *Arenaviridae*. The data show that the mechanism of inhibition is through specific ion chelation and not through steric hindrance or competition with the substrate for the active site. Further *in vivo* and screening experiments are needed to verify that ALD analogs or Mn^2+^ chelators could be used as lead molecules for the development of an antiviral. Nevertheless, our study suggests that bisphosphonate could be a promising scaffold that can be optimized to develop improved inhibitors of the 3′–5′ exonucleases of arenaviruses.

## Supplementary Material

PDB reference: Mopeia virus exonuclease domain, complexed with manganese, 6sx8

PDB reference: soaked with alendronate for 6 min, 6t6l

PDB reference: soaked with alendronate for 12 min, 6t2a

PDB reference: soaked with alendronate overnight, 6sy8

Supplementary Tables and Figures. DOI: 10.1107/S2052252522005061/mf5062sup1.pdf

## Figures and Tables

**Table 1:** Data processing, structure solution and refinement of MOPV NP-exo Values in parentheses are for the highest resolution shell.

		Soaked with ALD		
	Complexed with Mn^2+^	6 min	12 min	Overnight
Data processing				
Wavelength (Å)	0.9786	0.9724	0.972	0.9786
Space group	*P*121	*C*121	*P*121	*P*121
*a*, *b*, *c* (Å)	45.54, 38.01, 137.23	133.21, 111.34, 49.15	45.59, 37.97, 137.38	45.85, 38.29, 137.31
α, β, γ (°)	90.00, 92.94, 90.00	90.00, 104.17, 90.00	90.00, 93.31, 90.00	90.00, 92.63, 90.00
Resolution range (Å)	38.82–1.80 (1.864–1.800)	44.66–1.76 (1.82–1.76)	42.48–2.00 (2.07–2.00)	33.13–2.08 (2.16–2.08)
Total No. of reflections	159861 (15626)	242240 (24702)	90988 (6843)	154341 (15620)
No. of unique reflections	43794 (4304)	67897 (6824)	29975 (2211)	28850 (2855)
Completeness (%)	98.69 (97.97)	98.27 (98.88)	92.75 (69.22)	98.88 (98.40)
Multiplicity	3.7 (3.6)	3.6 (3.6)	3.0 (3.1)	5.3 (5.5)
〈*I*/σ(*I*)〉	14.99 (2.99)	10.54 (2.31)	23.73 (6.53)	12.85 (2.28)
*R*_meas_	0.04673 (0.4050)	0.08419 (0.5895)	0.03108 (0.1421)	0.1177 (0.9836)
CC_1/2_	0.999 (0.976)	0.996 (0.800)	0.999 (0.995)	0.998 (0.950)
Wilson *B* factor (Å^2^)	27.2	21.79	18.4	35.3
Structure solution and refinement				
No. of reflections, working set	43526 (4243)	67834 (6822)	29970 (2208)	28680 (2832)
No. of reflections, test set (%)	2195 (217)	3468 (330)	1983 (142)	1346 (124)
Reflections in test set (%)	5.0	5.21	6.6	4.7
*R*_cryst_	0.21	0.1908 (0.2394)	0.23	0.25
*R*_free_	0.25	0.2161 (0.2817)	0.27	0.28
No. of non-H atoms				
Total	3417	5721	3282	3302
Protein	3183	4926	3151	3183
Ligand	4	6	11	2
Water	230	789	120	117
R.m.s. deviations				
Bond lengths (Å)	0.007	0.009	0.007	0.014
Angles (°)	1.18	1.38	0.81	1.61
Average *B* factors (Å^2^)				
Overall	43.00	29.27	25.97	53.84
Protein	43.76	28.03	25.39	53.96
Ligand	38.10	20.22	41.34	40.17
Water	47.04	37.11	39.87	50.75
Ramachandran plot				
Favored (%)	97.43	96.89	97.93	97.43
Allowed (%)	2.57	3.11	2.07	2.57
Outliers (%)	0.00	0.00	0.00	0.00
Clashcore	6.77	6.72	6.83	4.10
PDB code	6sx8	6t6l	6t2a	6sy8
