# Supplementary material for: Inhibition of Arenaviridae nucleoprotein exonuclease by bisphosphonate
Source: IUCrJ. 2022 May 28;9(Pt 4):468–79. doi: 10.1107/S2052252522005061 (PMC9252148; doi:10.1107/S2052252522005061)
Supplement: Supplementary file 1 [file m-09-00468-sup1.pdf]

IUCrJ

Volume 9 (2022)

Supporting information for article:

Inhibition of *Arenaviridae* nucleoprotein exonuclease by bisphosphonate

Thi Hong Van Nguyen, Elsie Yekwa, Barbara Selisko, Bruno Canard, Karine Alvarez and François Ferron

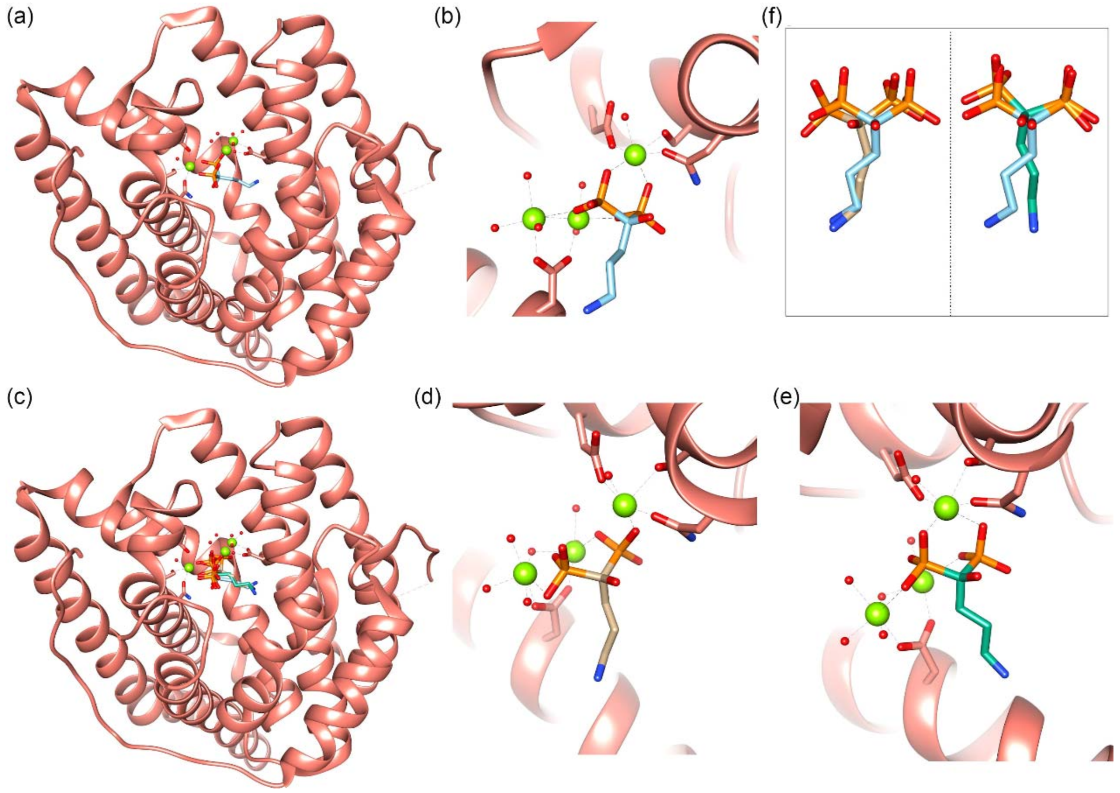

Figure S1 Geosmin synthase original structure versus exploration by Autodock Vina. (a) Crystal structure of geosmin synthase from Streptomyces coelicolor (ScGS) in complex with three Mg^{2+} ions and ALD (PDB:5DZ2). The protein structure is represented as a ribbon diagram with active site residues coordinating the Mg^{2+} ions (green spheres) shown in sticks. (b) Zoom of the observed coordination of Mg^{2+} ions by ALD in (PDB:5DZ2) (c) Resulting docking positions of ALD in the crystal structure of ScGS in complex with three Mg^{2+} ions with AutoDock Vina (beige and green molecules) (d and e) Zoom of the observed coordination of Mg^{2+} ions by ALD resulting from the docking (F) Superposition of the observed ALD (cyan) versus the resulting docking positions (beige and green).

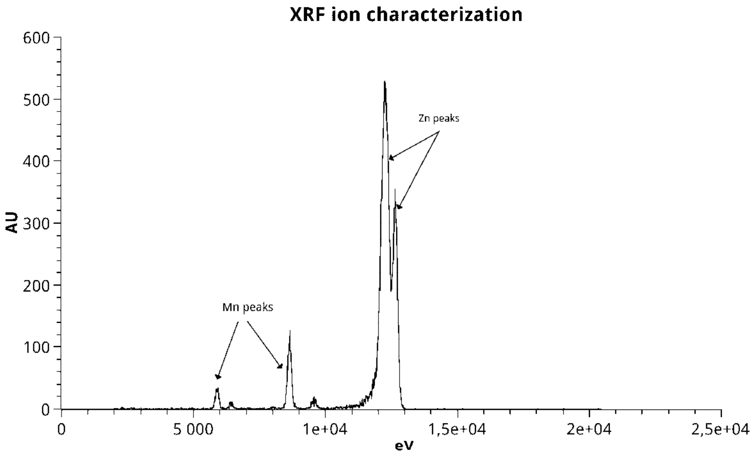

Figure S2 X-ray fluorescence scan to confirm the presence of Mn^{2+}. Scan realized on Proxima 1 synchrotron beam-line directly on the exposed crystal. It shows the presence of peaks corresponding to Mn^{2+} and Zn^{2+}.

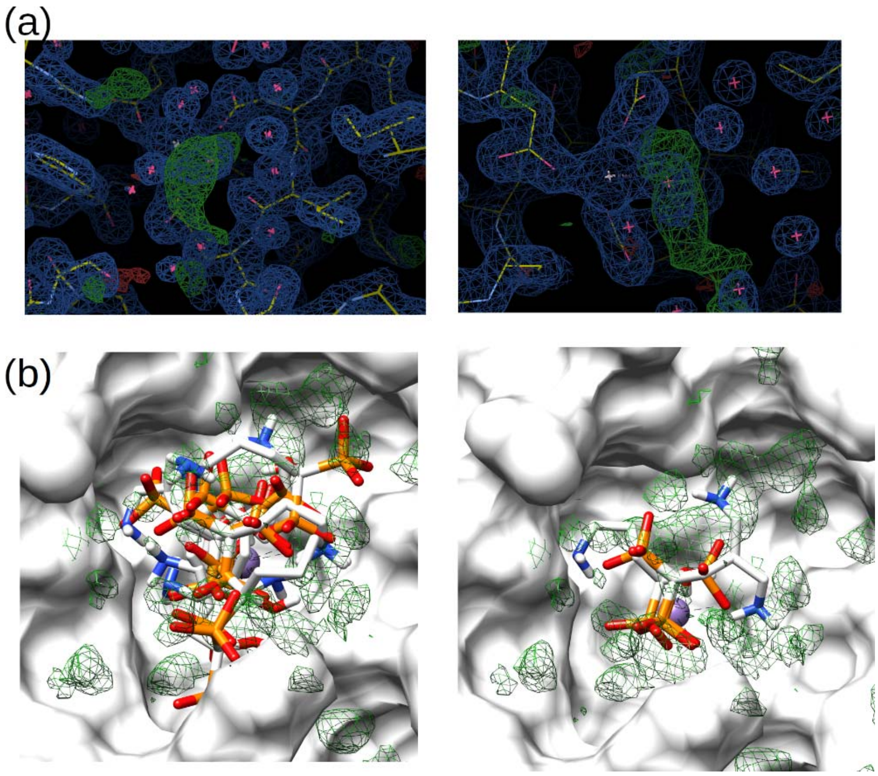

Figure S3 Use of experimental data for best pose selection. (a) Observed positive density (green) of FO-FC map spiking around the ion during refinement of soaked ALD (6min). As this density only appears in soaked crystals we assume that it is ghost signal of ALD and used that density to choose the best pose proposed by the docking experiment. The left panel is a view of the overall catalytic site; the right panel is the a zoomed view rotated by 45° from the left panel and centered on the Mn^{2+} ion. (b) Left : All poses resulting from the docking experiment (-4.2 to -4.7 kcal/mol). Right: Selected 3 poses compatible with the observed density (-4.6 kcal/mol).
